# Testing lipid markers as predictors of all-cause morbidity, cardiac disease, and mortality risk in captive western lowland gorillas (*Gorilla gorilla gorilla*)

**DOI:** 10.5194/pb-7-41-2020

**Published:** 2020-12-17

**Authors:** Ashley N. Edes, Janine L. Brown, Katie L. Edwards

**Affiliations:** 1 Center for Species Survival, Smithsonian Conservation Biology Institute, 1500 Remount Rd., Front Royal, VA 22630, USA; 2 North of England Zoological Society, Chester Zoo, Upton by Chester, CH2 1LH, UK; a currently at: Department of Reproductive and Behavioral Sciences, Saint Louis Zoo, St. Louis, MO 63110, USA

## Abstract

Great apes and humans develop many of the same health conditions, including cardiac disease as a leading cause of death. In humans, lipid markers are strong predictors of morbidity and mortality risk. To determine if they similarly predict risk in gorillas, we measured five serum lipid markers and calculated three lipoprotein ratios from zoo-housed western lowland gorillas (aged 6–52 years, n=61, subset with routine immobilizations only: n=47): total cholesterol (TC), triglycerides (TGs), high-density lipoprotein (HDL), low-density lipoprotein (LDL), apolipoprotein A1 (apoA1), TC/HDL, LDL/HDL, and TG/HDL. We examined each in relation to age and sex, then analyzed whether they predicted all-cause morbidity, cardiac disease, and mortality using generalized linear models (GLMs). Older age was significantly associated with higher TG, TC/HDL, LDL/HDL, and TG/HDL, and lower HDL and apoA1. With all ages combined, compared to females, males had significantly lower TG, TC/HDL, LDL/HDL, and TG/HDL, and higher HDL. Using GLMs, age, sex, and lower LDL/HDL were significant predictors of all-cause morbidity; this is consistent with research demonstrating lower LDL in humans with arthritis, which was the second most prevalent condition in this sample. In contrast to humans, lipid markers were not better predictors of cardiac disease and mortality risk in gorillas, with cardiac disease best predicted by age and sex alone, and mortality risk only by age. Similar results were observed when multimodel inference was used as an alternative analysis strategy, suggesting it can be used in place of or in addition to traditional methods for predicting risk.

## 1 Introduction

Great apes in zoos develop many chronic conditions that also are observed in humans, chief among them being heart disease. As it is for human populations around the globe, cardiac disease is the leading cause of death for captive great apes (Baitchman et al., 2006; Lowenstine et al., 2016; McManamon and Lowenstine, 2012; Murphy et al., 2018; Schmidt et al., 2006; Strong et al., 2016, 2017, 2018; Varki et al., 2009; Videan et al., 2009), affecting approximately 45 % of bonobos (*Pan paniscus*), 41 % of gorillas (*Gorilla gorilla gorilla*), 38 % of chimpanzees (*Pan troglodytes*), and 20 % of orangutans (*Pongo* spp.) in North American zoos (Lowenstine et al., 2016). Among gorillas, males are 8 times more likely to die of heart disease than females (Strong et al. 2018); it is the cause of death for 70 % of adult males (i.e., silverbacks) aged 30 years or older (Lowenstine et al., 2016; McManamon and Lowenstine, 2012). Given its prevalence, efforts continue to identify biological indicators that can predict risk and monitor progression of cardiac disease in gorillas and other great apes, such as leptin and adiposity (Dennis et al., 2019), blood pressure (Junge et al., 1998; McManamon and Lowenstine, 2012; Murphy et al., 2011, 2018), brain natriuretic peptide (BNP; McManamon and Lowenstine, 2012; Murphy et al., 2018; Murray et al., 2019), and inflammatory cytokines (Edes and Brand, 2020).

Humans are most commonly diagnosed with coronary artery disease (CAD), which is characterized by the accumulation of atherosclerotic plaques within arterial walls (Lowenstine et al., 2016; Strong et al., 2018; Varki et al., 2009). As such, hyperlipidemia is a well-known risk factor for CAD in humans, although half of all coronary events occur in people without it (Libby, 2002; Willerson and Ridker, 2004). Given the similarities in disease prevalence between humans and gorillas, it has generally been assumed that the two species would share similar risk factors (e.g., Benirschke and Adams, 1980; Junge et al., 1998). Seemingly consistent with this expectation, zoo-housed gorillas have lipid profiles that appear proatherogenic (i.e., might promote the growth of atherosclerotic plaques) and would be considered hyperlipidemic if observed in humans (Baitchman et al., 2006; Benirschke and Adams, 1980; Crissey et al., 1999; Kenny et al., 1994; McGuire et al., 1989; Murphy et al., 2018; Popovich et al., 1997; Schmidt et al., 2006; Varki et al., 2009). Furthermore, total cholesterol and triglycerides are higher in captive than free-ranging gorillas (Schmidt et al., 2006), who seem to rarely develop cardiac disease (Lowenstine et al., 2016). Although atherosclerotic plaques (Cousins, 1972; Hruban et al., 1986; Janssen and Bush, 1990; Kenny et al., 1994; McManamon and Lowenstine, 2012) and even CAD (Murphy et al., 2011) have been documented in a few individuals, severe atherosclerosis in gorillas and other great apes is rare (Lowenstine et al., 2016; McManamon and Lowenstine, 2012; Varki et al., 2009).

Instead, gorillas have primarily been diagnosed with fibrosing cardiomyopathy (FCM), which is characterized by an increase in fibrous connective tissue in the heart, resulting in reduced contractility and conduction (Lowenstine et al., 2016; McManamon and Lowenstine, 2012; Murphy et al., 2018; Strong et al., 2018). Similar conditions, also without atherosclerosis, occur in humans (e.g., idiopathic myocardial fibrosis; Lowenstine et al., 2016). The second most common form of cardiac disease in captive western lowland gorillas is aortic dissection (Kenny et al., 1994; Lowenstine et al., 2016; McManamon and Lowenstine, 2012; Murphy et al., 2018; Strong et al., 2018), although it has only been documented in males (Strong et al., 2018). While aortic dissections can occur along ruptured plaques in humans, this was not observed in a study of aortic dissection in eight gorillas even though six were reported to have atherosclerosis (Kenny et al., 1994).

Given the low prevalence of atherosclerosis reported at necropsy, in one of the first reviews on causes of disease and mortality in gorillas, Benirschke and Adams (1980) reported that it was “of minor importance” to cardiac disease risk. Citing this paper, some have extrapolated that there are no correlations between lipid profiles and cardiac disease in gorillas (e.g., Baitchman et al., 2006; Junge et al., 1998), yet to our knowledge, there are no published studies examining these relationships. Zoos frequently measure lipid markers such as total cholesterol and triglycerides as part of routine health monitoring in great apes and other species, although the data are infrequently published, in part because of the availability of the Species360 database (Species360, 2020) that is widely used by zoo professionals and veterinarians and integrated into medical records software. Means and standard deviations have been published for some of the lipid markers included herein in captive gorillas (Table 1), with total cholesterol being the most common. Of the few published studies, only one analyzed age and sex associations, and the authors reported no significant associations between total cholesterol and either variable (McGuire et al., 1989).

**Table 1:** Descriptive statistics for lipid markers in western lowland gorillas reported in previous studies and from the Species360 database.

	TC	TG	HDL	LDL	apoA1	TC/	LDL/	TG/	
	(mmol/L)	(mmol/L)	(mmol/L)	(mmol/L)	(mg/dL)	HDL	HDL	HDL	Source
$\bar{x}$	8.70 all								${}^{a}$ McClure et al. (1972)
	8.59 M								n = 15, 5 males
	8.76 F								
SD	1.26 all								
	1.35 M								
	1.21 F								
min	6.36 all								
	6.36 M								
	6.72 F								
max	11.77 all								
	11.77 M								
	11.74 F								
$\bar{x}$	7.27								${}^{a}$ McGuire et al. (1989)
SD	0.31								n = 59, sex distribution
min	2.79								not reported
max	10.91								
$\bar{x}$									${}^{a}$ Junge et al. (1998)
SD									n=5, all males
min	5.02								
max	7.91								
$\bar{x}$	6.25	1.27	2.15	3.70		*2.91*	*1.72*	*0.59*	${}^{a,b}$ Crissey et al. (1999)
SD	0.37	0.01	0.16	0.33					n = 25, sex distribution
min									not reported
max									
$\bar{x}$	6.78	1.46	2.15	3.97	266	3.6	*1.85*	*0.68*	${}^{b}$ Baitchman et al. (2006)
SD	1.67	0.89	1.04	1.01	76	1.0			n = 15, 5 males
min									
max									
$\bar{x}$	6.61 M	1.43 M							${}^{a}$ Dennis et al. (2019)
	5.63 F	1.83 F							n = 69, 44 males
SD	1.65 M	0.72 M							
	1.71 F	0.95 F							
min	3.70 M	0.44 M							
	3.67 F	0.75 F							
max	9.75 M	4.23 M							
	9.90 F	4.17 F							
$\bar{x}$	6.4	1.3	2.22			*2.88*		*0.59*	${}^{b}$ Species360
SD									TC n = 1871 (403 animals)
min	0 (3.5)	0 (0.4)	0.3 (0.66)						TG n = 1083 (300 animals)
max	13.9 (11.2)	4.0 (3.1)	6.53 (4.38)						HDL n = 267 (125 animals)

${}^{a}$ Reported TC, HDL, LDL, and TG values have been converted to mmol/L (Rugge et al. 2011) for ease of comparison to values presented herein. ${}^{b}$ Italicized lipoprotein ratios not reported, calculated here for comparison to lipoprotein ratios presented herein. ${}^{c}$ Values in parentheses are upper and lower boundaries of calculated reference intervals (only values from serum collected during immobilizations for routine veterinary examinations are included in reference intervals). apoA1 – apolipoprotein A1; F – females; HDL – high-density lipoprotein; LDL – low-density lipoprotein; M – males; TC – total cholesterol; TG – triglycerides.

As biomarker thresholds are often used as an indicator of poor health (e.g., hypertension, hypoglycemia), setting diagnostic criteria first requires knowledge of age- and sex-related patterns, yet there are a lack of even basic biological data on lipids in gorillas. Therefore, we measured five lipid markers from serum samples collected during immobilizations and calculated three lipoprotein ratios to examine relationships with sex and age as well as to determine if these physiological indicators predict risk of all-cause morbidity, cardiac disease, and mortality in gorillas at three North American zoos. We do want to emphasize that these data are presented for comparative purposes and are not intended to define reference ranges or healthy versus clinical levels of circulating lipid markers and lipoprotein ratios, and as such any reported differences may not be clinically meaningful.

## 2 Methods

### 2.1 Subjects

We obtained cryopreserved serum samples that had been collected during immobilizations from western lowland gorillas (n = 61) currently or previously housed at the Columbus Zoo and Aquarium (males: n = 10; females: n = 16), Louisville Zoo (males: n = 9; females: n = 12), and Omaha's Henry Doorly Zoo (males: n = 10; females: n = 4). One sample was analyzed per gorilla. Males ranged in age from 7–52 years (n = 29, $\bar{x}$ = 22.00, SD = 10.90) and females from 6–52 years (n = 32, $\bar{x}$ = 23.53, SD = 14.40). This study complied with The Ohio State University Animal Care and Use (IACUC) protocols, was approved by each participating institution, and adhered to the American Society of Primatologists' Principles for the Ethical Treatment of Non-Human Primates.

### 2.2 Lipid markers and health data

Five lipid markers were assayed for each gorilla: apolipoprotein A1 (apoA1), high-density lipoprotein (HDL), low-density lipoprotein (LDL), total cholesterol (TC), and triglycerides (TGs). These data were also used to calculate TC/HDL, LDL/HDL, and TG/HDL ratios. TG, TC, HDL, LDL, and apoA1 were quantitated on a RX Daytona automated clinical chemistry analyzer (Randox Laboratories-US, Ltd., Kearneysville, WV, USA). Commercially available reagents (TR3823, CH3810, CH3811, CH3841, and LP3838, respectively), calibrators (CAL2351 (TG and TC), CH2673 (HDL and LDL), LP3023 (apoA1)), and two-level controls (HN1530 and HE1532 (TG and TC), LE2661 and LE2663 (HDL, LDL and apoA1)) were all purchased from Randox Laboratories-US, Ltd. (Kearneysville, WV, USA). The technical ranges were 0–12.8 mmol/L, 0–17.0 mmol/L, 0.189–3.73 mmol/L, 0.189–22.2 mmol/L, and 6.5–233.0 mg/dL, respectively. Serum was generally run neat or diluted 1:5 with saline (SA3854) where necessary. The analyzer was subject to routine quality control measurements throughout the study, with normal and elevated controls for each analyte maintained within 2 standard deviations (SD) of the respective target value. Diagnosed chronic conditions, including cardiac disease, and age at death (if applicable) were recorded from zoo medical records. Chronic conditions, cardiac disease, and mortality were coded as dichotomous 0,1 variables (disease: 1 = present; mortality: 1 = deceased).

### 2.3 Quantitative analyses

Some studies have shown fluctuations in lipids and lipoproteins in response to acute stressors in humans (Bachen et al., 2002; Dimsdale and Herd, 1982; Niaura et al., 1992; Steptoe and Brydon, 2005; Stoney et al., 1988; van Doornen et al., 1998), although results vary widely between individuals and based on context (e.g., laboratory versus real-life stressors). Serum samples from 47 of the 61 gorillas were obtained during immobilizations for routine health exams. The other samples were collected during immobilizations for clinical events (e.g., illnesses such as respiratory infections, injuries requiring veterinary treatment, medical interventions during parturition) rather than routine exams. Because these clinical events could impact lipid and lipoprotein levels in gorillas the same way acute stressors do in humans, we present results for the whole dataset as well as for samples collected during routine immobilizations only (n = 47).

First, we examined effects of age and sex on serum lipid profiles. HDL and apoA1 were normally distributed and analyzed using linear regression; TC, TG, LDL, TC/HDL, LDL/HDL, and TG/HDL had a positive skew and were analyzed using generalized linear models (GLMs) with a gamma distribution and a log-link function. We then analyzed whether lipid markers predicted risk of all-cause morbidity, cardiac disease, and mortality using GLMs with a binomial distribution and a logit link. Our baseline model contained just age and sex, as these variables independently associate with many health outcomes. Eight additional models were constructed, each one containing age, sex, and one lipid marker. For comparative purposes, we also analyzed differences between gorillas with and without chronic conditions, both altogether and cardiac disease only, using either linear regression or GLM based on the distribution of each biomarker. Zoo ID was not included as a random effect for any models, as it did not improve models and often resulted in overfitting. Significance was set at α=0.05. However, given the exploratory nature of this study, the small sample size, and because statistical significance does not always indicate biological importance, we also discuss results at p ≤ 0.10 as nearing significance.

While these quantitative methods are routinely used to analyze physiological data, null hypothesis significance testing has been criticized frequently (e.g., Greenland et al., 2016; Nakagawa and Cuthill, 2007; Smith, 2018) and could be a problematic approach given that p values do not equate to biological or clinical importance. One information theoretic approach that has been proposed to overcome these issues in ecological studies is multimodel inference. Therefore, we wanted to investigate multimodel inference as an alternative method for identifying biomarkers that may be useful in predicting and/or monitoring disease risk and progression. To do this, we next analyzed the risk models described above as well as a global model (age and sex plus all eight lipid markers) using multimodel inference to determine which combination of variables best explain variation in each health outcome (Grueber et al., 2011; Harrison et al., 2018; Symonds and Moussalli, 2011). Multimodel inference uses Akaike's information criterion (AIC/AICc for small sample sizes) and model weights, which is the probability that a given model is the best of those included, to rank models relative to one another. While there is some debate over criterion for a top model set, one suggestion is models with ΔAICc < 2 are equivalent to the top ranked model and that models with ΔAICc < 6 should not be discounted, especially in predictive contexts (Arnold, 2010; Harrison et al., 2018; Richards, 2005, 2008; Richards et al., 2011; Symonds and Moussalli, 2011). Therefore, we present the top model as well as the top model set, which contains all models with ΔAICc < 6 and weights summing to ≥ 0.95 (Harrison et al., 2018). An evidence ratio (ER) also is calculated for each model, which indicates the likelihood of the top model being better than the current model (e.g., ER = 2.5 means the top model is approximately 2.5 times more likely to be the best model than the model to which it is being compared; Symonds and Moussalli, 2011). Finally, we calculated Nagelkerke's adjusted $R^{2}$ as an estimate of the variation in risk explained by each model.

Statistical analyses were conducted using R (v3.5.0, R Core Team 2018). GLMs were analyzed using the “lme4” package (Bates et al., 2019). Multimodel inference was conducted using “MuMin” (Bartoń, 2019), with Nagelkerke's adjusted $R^{2}$ estimated using “rcompanion” (Mangiafico, 2020). Visualizations were made with “ggplot2” (Wickham et al., 2020).

## 3 Results

More than half of the gorillas in this sample, 55.7 % (n = 34), were diagnosed with a chronic condition of some kind, and 31.1 % (n = 19) died between sample collection and data analysis. Cardiac disease was diagnosed in 42.6 % (n = 26) of gorillas. Other diagnosed conditions included arthritis, obesity, hypothyroidism, hypertension, cancer, and periodontal disease; at least nine individuals (14.8 %) had comorbidities. Males were more likely than females to have been diagnosed with a poor health outcome, with 72.4 % (n = 21) having at least one chronic condition and 65.5 % (n = 19) having cardiac disease; 31 % (n = 9) of males have died since sample collection. Among females, 40.6 % (n = 13) had one or more chronic conditions, 21.9 % (n = 7) had cardiac disease, and 31.3 % (n = 10) are now deceased.

**Figure 1:**
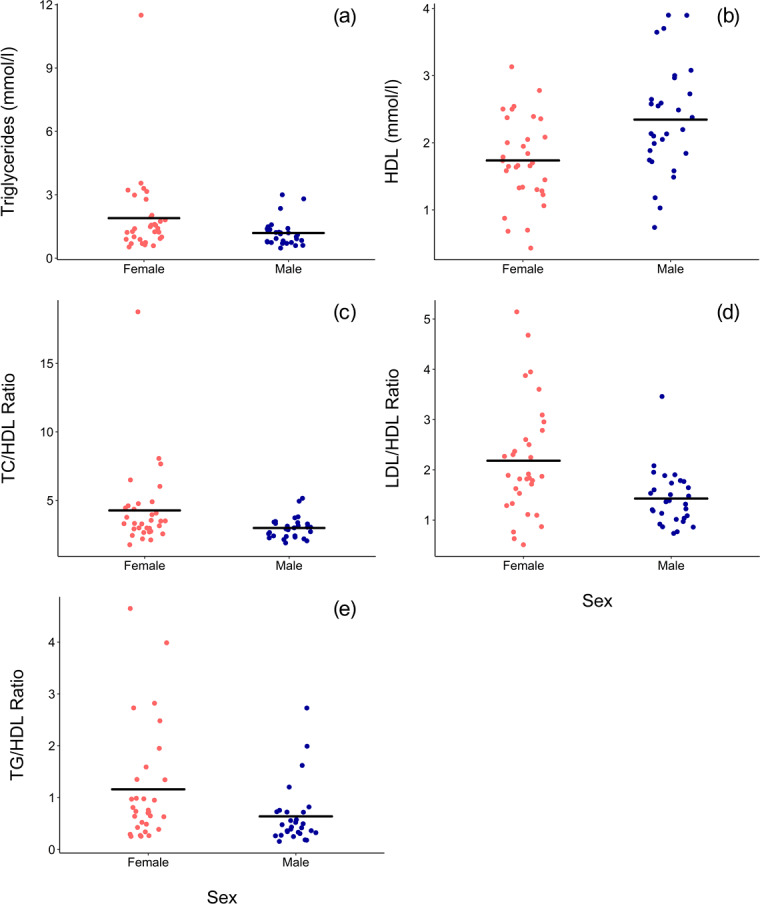
In a sample of zoo-housed western lowland gorillas (n = 61), males have significantly **(a)** lower TG (β ± SE = -0.405 ±  0.187, p = 0.035), **(b)** higher HDL (β ± SE = 0.576 ± 0.176, p = 0.002), **(c)** lower TC/HDL ratio (β ± SE = -0.314 ±  0.122, p = 0.013), **(d)** lower LDL/HDL ratio (β ± SE = -0.414 ± 0.115, p = 0.001), and **(e)** lower TG/HDL ratio (β ± SE =  -0.881 ± 0.387, p=0.027; to improve visualization, figure shown without one outlier value of 26.7, which does not change results) than females. HDL – high-density lipoprotein; LDL – low-density lipoprotein; TC – total cholesterol; TG – triglycerides.

**Figure 2:**
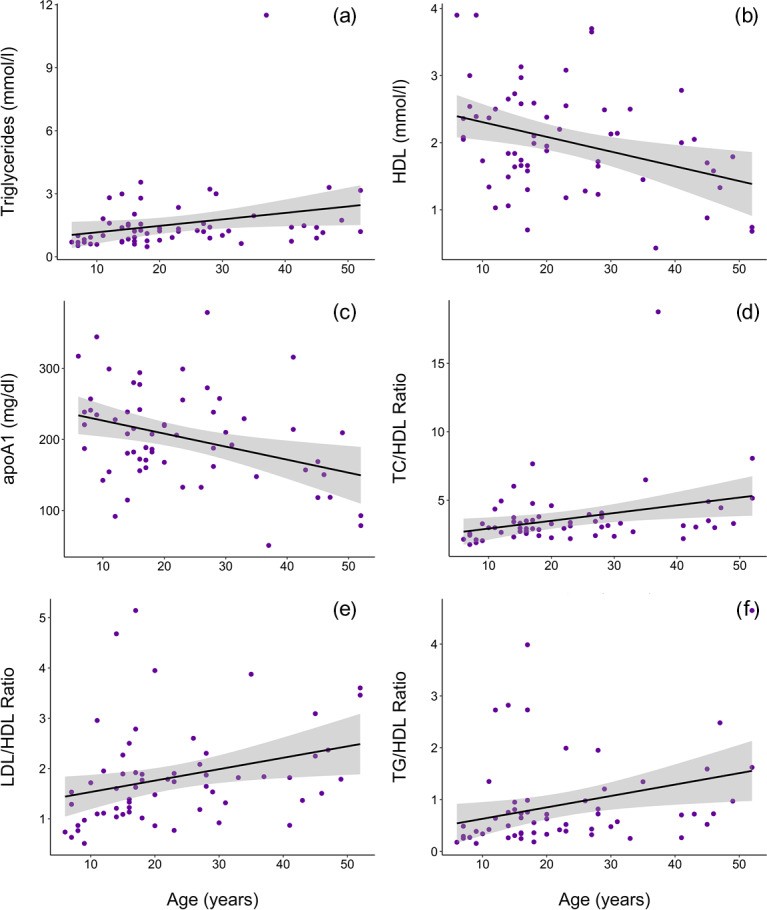
In a sample of zoo-housed western lowland gorillas (n = 61), older animals have significantly **(a)** higher TG (β ± SE = 0.019±0.007), **(b)** lower HDL (β ± SE = -0.021 ± 0.007, p = 0.004), **(c)** lower apoA1 (β ± SE = -1.772 ± 0.620, p = 0.006), **(d)** higher TC/HDL ratio (β ± SE = 0.014 ± 0.005, p = 0.006), **(e)** higher LDL/HDL ratio (β ± SE = 0.012 ± 0.005, p =  0.011), and **(f)** higher TG/HDL ratio (β ± SE = 0.038 ± 0.015, p = 0.015; to improve visualization, figure shown without one outlier value of 26.7, which does not change results) compared to younger conspecifics. apoA1 – apolipoprotein A1; HDL – high-density lipoprotein; LDL – low-density lipoprotein; TC – total cholesterol; TG – triglycerides.

Ranges, means, and standard deviations for each lipid marker for all samples and for samples from routine immobilizations only, with both males and females combined and separately by sex, are presented in Table 2. Associations of sex (β ± SE = 0.062 ± 0.066, p = 0.356) and age (β ± SE = -0.001 ± 0.003, p = 0.653) with cholesterol were not significant. Males had significantly lower TG than females when all values were retained in the dataset (β ± SE = -0.405 ± 0.187, p = 0.035, Fig. 1a) but not when only routine immobilizations were included (β ± SE = -0.189 ± 0.150, p = 0.214). Older gorillas had significantly higher circulating levels of TG than younger conspecifics (β ± SE = 0.019 ± 0.007, p = 0.014, Fig. 2a). HDL was significantly higher among males (β ± SE = 0.576 ± 0.176, p = 0.002, Fig. 1b) and inversely associated with age (β ± SE = -0.021 ± 0.007, p = 0.004, Fig. 2b). Associations of LDL with sex (β ± SE = -0.076 ± 0.108, p = 0.483) and age (β ± SE = 0.001 ± 0.004, p = 0.895) were not significant. Unlike HDL, apoA1 was not significantly different between males and females (β ± SE = 25.539 ± 15.734, p = 0.110) but showed the same inverse association with age (β ± SE = -1.772 ± 0.620, p = 0.006, Fig. 2c). When only samples from routine immobilizations were included, apoA1 was no longer significantly associated with age (β ± SE = -1.332 ± 0.685, p = 0.058). All three ratios were significantly lower in males (TC/HDL: β ± SE = -0.314 ±  0.122, p = 0.013, Fig. 1c; LDL/HDL: β ± SE = -0.414 ± 0.115, p = 0.001, Fig. 1d, TG/HDL: β ± SE = -0.881 ± 0.387, p = 0.027, Fig. 1e) and were positively associated with age (TC/HDL: β ± SE = 0.014 ± 0.005, p = 0.006, Fig. 2d; LDL/HDL: β ± SE = 0.012 ± 0.005, p = 0.011, Fig. 2e; TG/HDL: β ±  SE = 0.038 ± 0.015, p = 0.015, Fig. 2f).

**Table 2:** Descriptive statistics for total cholesterol, triglycerides, HDL, LDL, apolipoprotein A1, TC/HDL ratio, LDL/HDL ratio, and TG/HDL ratio in a sample of zoo-housed western lowland gorillas.

	All					Females only				
	n	$\bar{x}$	SD	min	max	n	$\bar{x}$	SD	min	max
All values										
TC (mmol/L)	61	6.34	1.63	3.81	12.61	32	6.14	1.51	4.15	10.91
TG (mmol/L)	61	1.56	1.51	0.48	11.50	32	1.89	1.96	0.53	11.50
HDL (mmol/L)	61	2.03	0.78	0.43	3.90	32	1.74	0.64	0.43	3.13
LDL (mmol/L)	61	3.24	1.36	0.79	7.83	32	3.36	1.53	0.79	7.83
apoA1 (mg/dl)	61	203.03	65.88	50.87	378.56	32	189.59	60.21	50.87	315.74
TC/HDL	61	3.65	2.34	1.76	18.77	32	4.26	3.04	1.76	18.77
LDL/HDL	61	1.82	0.97	0.51	5.14	32	2.18	1.13	0.51	5.14
TG/HDL	61	1.33	3.43	0.16	26.74	32	1.96	4.65	0.25	26.74
Routine immobilizations only										
TC (mmol/L)	47	6.37	1.65	3.98	12.61	25	6.02	1.47	4.15	10.91
TG (mmol/L)	47	1.30	0.72	0.48	3.55	25	1.40	0.83	0.53	3.55
HDL (mmol/L)	47	2.20	0.73	0.68	3.90	25	1.89	0.55	0.68	3.13
LDL (mmol/L)	47	3.26	1.38	1.22	7.83	25	3.35	1.55	1.22	7.83
apoA1 (mg/dl)	47	218.30	60.20	92.73	378.56	25	204.04	53.59	92.73	315.74
TC/HDL	47	3.09	1.01	1.76	8.06	25	3.42	1.23	1.76	8.06
LDL/HDL	47	1.61	0.74	0.51	3.95	25	1.91	0.86	0.51	3.95
TG/HDL	47	0.74	0.77	0.16	4.65	25	0.92	0.96	0.25	4.65
						Males only				
						n	$\bar{x}$	SD	min	max
All values										
TC (mmol/L)						29	6.55	1.75	3.81	12.61
TG (mmol/L)						29	1.19	0.61	0.48	3.00
HDL (mmol/L)						29	2.34	0.82	0.74	3.90
LDL (mmol/L)						29	3.11	1.16	1.80	7.60
apoA1 (mg/dl)						29	217.85	69.67	78.71	378.56
TC/HDL						29	2.98	0.76	1.89	5.15
LDL/HDL						29	1.43	0.55	0.74	3.46
TG/HDL						29	0.64	0.58	0.16	2.73
Routine immobilizations only										
TC (mmol/L)						22	6.77	1.79	3.98	12.61
TG (mmol/L)						22	1.77	0.58	0.48	3.00
HDL (mmol/L)						22	2.56	0.76	1.18	3.90
LDL (mmol/L)						22	3.15	1.18	1.96	7.60
apoA1 (mg/dl)						22	234.51	64.33	132.76	378.56
TC/HDL						22	2.72	0.49	1.89	3.45
LDL/HDL						22	1.27	0.37	0.74	2.08
TG/HDL						22	0.54	0.42	0.16	1.99

apoA1 – apolipoprotein A1; HDL – high-density lipoprotein; LDL – low-density lipoprotein; TC – total cholesterol; TG – triglycerides.

We next analyzed how models containing sex, age, and individual lipid markers predicted all-cause morbidity, cardiac disease, and mortality (Table 3). In the baseline model, all-cause morbidity risk was significantly higher in male gorillas (β ± SE = 1.988 ± 0.704, p = 0.005) and increased with age (β ± SE = 0.132 ± 0.037, p < 0.001). While sex and age remained significant predictors in nearly all models containing sex, age, and one lipid marker, no individual lipid markers were significantly associated with all-cause morbidity risk, although LDL/HDL ratio neared statistical significance when only routine immobilizations were considered (β ± SE = -1.664 ± 0.958, p = 0.082). As with all-cause morbidity, cardiac disease risk was significantly higher in males (β ± SE = 2.175 ± 0.640, p = 0.001) and increased significantly with age (β ± SE = 0.050 ± 0.025, p = 0.048). In models containing a lipid marker as well as sex and age, only sex and age were significant predictors of cardiac disease risk. Older age was significantly associated with increased mortality risk (β ± SE = 0.062 ± 0.024, p = 0.008), but the effect of sex was not significant (β ± SE = 0.147 ± 0.599, p = 0.806). In models containing lipid markers as well as sex and age, only age was significantly associated with mortality risk.

**Table 3:** Predictions of all-cause morbidity, cardiac disease, and mortality risk in zoo-housed western lowland gorillas from generalized linear models (GLMs) containing age and sex only as well as models containing age, sex, and individual lipid markers. Bold values are significant at p≤0.05.

	All-cause morbidity			Cardiac disease			Mortality		
Biomarkers	β	SE	p	β	SE	p	β	SE	p
All values retained (n=61)									
Age Sex	0.132 1.988	0.037 0.704	<0.001**0.005**	0.050 2.175	0.025 0.640	**0.0480.001**	0.062 0.147	0.024 0.599	**0.008** 0.806
TC (mmol/L) Age Sex	-0.143 0.135 2.067	0.216 0.038 0.721	0.508 <0.001**0.004**	0.191 0.052 2.153	0.199 0.025 0.648	0.339 **0.0400.001**	0.247 0.067 0.083	0.182 0.024 0.610	0.175 **0.006** 0.892
TG (mmol/L) Age Sex	0.040 0.130 2.014	0.247 0.037 0.722	0.872 **0.0010.005**	0.178 0.045 2.313	0.204 0.026 0.666	0.384 **0.0840.001**	0.276 0.057 0.325	0.279 0.024 0.623	0.322 **0.019** 0.601
HDL (mmol/L) Age Sex	0.018 0.132 1.977	0.481 0.037 0.767	0.971 <0.001**0.010**	0.284 0.055 2.024	0.435 0.026 0.676	0.514 **0.0370.003**	0.155 0.065 0.058	0.441 0.025 0.652	0.726 **0.009** 0.929
LDL (mmol/L) Age Sex	-0.422 0.144 1.936	0.286 0.041 0.712	0.141 <0.001**0.007**	0.007 0.050 2.177	0.234 0.025 0.644	0.977 **0.0480.001**	0.243 0.064 0.232	0.210 0.024 0.613	0.249 **0.007** 0.705
apoA1 (mg/dl) Age Sex	-0.003 0.129 2.074	0.006 0.037 0.729	0.622 <0.001**0.004**	0.001 0.052 2.140	0.005 0.026 0.650	0.769 **0.0480.001**	0.002 0.066 0.096	0.005 0.025 0.613	0.685 **0.009** 0.875
TC/HDL Age Sex	0.017 0.131 2.008	0.161 0.038 0.728	0.916 **0.0010.006**	0.107 0.045 2.326	0.132 0.026 0.673	0.417 **0.0870.001**	0.251 0.054 0.446	0.220 0.025 0.649	0.255 **0.030** 0.491
LDL/HDL Age Sex	-0.760 0.152 1.478	0.568 0.043 0.758	0.181 <0.001**0.051**	-0.339 0.056 1.966	0.446 0.027 0.679	0.448 **0.0360.004**	0.356 0.058 0.447	0.345 0.025 0.685	0.303 **0.017** 0.514
TG/HDL	0.055	0.150	0.714	0.097	0.103	0.345	0.276	0.337	0.412
Age	0.129	0.037	**0.001**	0.045	0.026	**0.081**	0.055	0.025	**0.024**
Sex	2.038	0.714	**0.004**	2.312	0.660	**0.001**	0.377	0.636	0.553
Routine immobilizations only (n=47)									
Age Sex	0.116 2.211	0.039 0.790	**0.0030.005**	0.047 2.303	0.029 0.726	0.104 0.002	0.058 0.002	0.028 0.691	**0.036** 0.998
TC (mmol/L) Age Sex	-0.273 0.119 2.516	0.255 0.040 0.887	0.286 **0.0030.005**	0.032 0.047 2.281	0.227 0.029 0.740	0.888 0.103 **0.002**	0.246 0.062 -0.155	0.208 0.028 0.713	0.237 0.029 0.828
TG (mmol/L) Age Sex	-0.684 0.130 2.057	0.621 0.043 0.797	0.271 **0.0020.010**	-0.648 0.060 2.254	0.582 0.032 0.741	0.265 **0.0600.002**	-0.001 0.058 0.002	0.493 0.029 0.698	0.999 **0.042** 0.998
HDL (mmol/L) Age Sex	-0.015 0.115 2.223	0.622 0.040 0.924	0.981 **0.0040.016**	0.619 0.059 1.959	0.568 0.031 0.785	0.276 **0.0600.013**	0.532 0.068 -0.335	0.564 0.030 0.793	0.346 **0.023** 0.672

**Table 3:** Continued.

	All-cause morbidity			Cardiac disease			Mortality		
Biomarkers	β	SE	p	β	SE	p	β	SE	p
LDL (mmol/L) Age Sex	-0.537 0.132 2.281	0.353 0.044 0.829	0.128 **0.0030.006**	0.001 0.047 2.303	0.275 0.029 0.729	0.998 0.104 **0.002**	0.304 0.061 0.114	0.239 0.029 0.714	0.203 **0.035** 0.873
apoA1 (mg/dl) Age Sex	-0.000 0.115 2.227	0.007 0.039 0.836	0.955 **0.0030.008**	0.007 0.058 2.165	0.006 0.031 0.741	0.271 **0.0630.003**	0.004 0.064 -0.102	0.006 0.029 0.714	0.505 **0.029** 0.886
TC/HDL Age Sex	-0.605 0.142 1.813	0.494 0.050 0.858	0.221 **0.0040.034**	-1.035 0.075 1.870	0.658 0.034 0.767	0.116 **0.0290.015**	0.133 0.055 0.094	0.403 0.030 0.745	0.741 **0.067** 0.899
LDL/HDL Age Sex	-1.664 0.167 1.409	0.958 0.059 0.859	**0.0820.004** 0.101	-0.952 0.066 1.862	0.708 0.034 0.773	0.179 **0.0500.016**	0.329 0.055 0.228	0.553 0.029 0.798	0.552 **0.060** 0.775
TG/HDL	-0.643	0.577	0.265	-1.323	0.916	0.149	0.045	0.473	0.924
Age	0.134	0.046	**0.003**	0.070	0.033	**0.034**	0.058	0.030	**0.052**
Sex	2.003	0.818	**0.014**	2.072	0.757	**0.006**	0.019	0.714	0.979

apoA1 – apolipoprotein A1; HDL – high-density lipoprotein; LDL – low-density lipoprotein; TC – total cholesterol; TG – triglycerides.

Ranges, means, and standard deviations, as well as results from GLMs between lipid markers based on presence or absence of disease, are presented in Tables 4 and 5. Only two analyses neared statistical significance (α < 0.10) for all-cause morbidity; TG/HDL was higher in females with at least one chronic condition (β ± SE = 1.086 ± 0.571, p = 0.067; Table 4), and there was an inverse association between TC and all-cause morbidity in females when only routine immobilizations were included (β ± SE = -0.169 ± 0.095, p = 0.087; Table 4). When all values were retained, females with cardiac disease had significantly higher TG/HDL than females without (β ± SE = 1.240 ± 0.557, p = 0.034; Table 5), but this relationship was no longer significant when only routine immobilizations were included (β ± SE = -0.619 ± 0.481, p = 0.211; Table 5). When only routine immobilizations were analyzed, gorillas with cardiac disease neared significantly higher HDL (β ± SE = 0.405 ± 0.210, p = 0.060; Table 5) and had significantly lower TC/HDL (β ± SE = -0.186 ± 0.089, p = 0.042; Table 5), LDL/HDL (β ± SE = -0.269 ± 0.126, p = 0.038; Table 5), and TG/HDL (β ± SE = -0.539 ± 0.261, p = 0.044; Table 5) ratios.

**Table 4:** Differences in lipid markers and lipoprotein ratios in a sample of zoo-housed western lowland gorillas based on presence or absence of diagnosed chronic conditions, including cardiac disease.

	Present				Absent				Analysis		
	$\bar{x}$	SD	min	max	$\bar{x}$	SD	min	max	B	SE	p
Both sexes, all values retained (n=61)											
TC${}^{a}$	6.32	1.74	3.81	12.61	6.37	1.52	3.98	10.91	-0.008	0.067	0.906
TG${}^{a}$	1.68	1.88	0.48	11.50	1.41	0.88	0.53	3.55	0.171	0.241	0.480
HDL${}^{b}$	2.01	0.80	0.43	3.70	2.05	0.78	0.70	3.90	-0.038	0.204	0.853
LDL${}^{a}$	3.04	1.20	0.79	7.60	3.48	1.53	1.22	7.83	-0.135	0.107	0.210
apoA1${}^{b}$	195.60	71.21	50.87	378.56	212.38	58.46	114.63	344.29	-16.79	16.99	0.327
TC/HDL${}^{a}$	3.83	2.94	1.76	18.77	3.43	1.25	2.04	7.66	0.111	0.161	0.493
LDL/HDL${}^{a}$	1.72	0.81	0.63	3.88	1.96	1.15	0.51	5.14	-0.129	0.135	0.344
TG/HDL${}^{a}$	1.65	4.52	0.19	26.74	0.93	0.96	0.16	3.99	0.574	0.557	0.307
Both sexes, routine immobilizations only (n=47)											
TC${}^{b}$	6.33	1.69	4.15	12.61	6.43	1.64	3.98	10.91	-0.016	0.077	0.840
TG${}^{a}$	1.27	0.62	0.48	3.16	1.34	0.85	0.53	3.55	-0.054	0.164	0.744
HDL${}^{b}$	2.24	0.71	0.68	3.70	2.15	0.77	1.18	3.90	0.091	0.217	0.677
LDL${}^{b}$	3.04	1.15	1.49	7.60	3.52	1.61	1.22	7.83	-0.146	0.122	0.237
apoA1${}^{b}$	218.31	61.32	92.73	378.56	218.29	60.29	132.69	344.29	0.020	17.86	0.999
TC/HDL${}^{a}$	3.03	1.16	1.76	8.06	3.17	0.81	2.04	4.76	-0.043	0.097	0.663
LDL/HDL${}^{b}$	1.47	0.63	0.63	3.60	1.79	0.85	0.51	3.95	-0.196	0.131	0.143
TG/HDL${}^{a}$	0.72	0.84	0.19	4.65	0.78	0.69	0.16	2.73	-0.076	0.311	0.808
Males, all values retained (n=29)											
TC${}^{a}$	6.60	1.85	3.81	12.61	6.43	1.56	3.98	8.35	0.025	0.113	0.825
TG${}^{a}$	1.26	0.61	0.48	3.00	0.99	0.60	0.60	2.35	0.239	0.216	0.278
HDL${}^{b}$	2.30	0.75	0.74	3.70	2.45	1.03	1.18	3.90	-0.148	0.345	0.672
LDL${}^{a}$	3.14	1.21	1.96	7.60	3.02	1.07	1.80	4.85	0.039	0.157	0.808
apoA1${}^{b}$	212.35	69.38	78.71	378.56	232.27	73.03	132.76	344.29	-19.92	29.23	0.501
TC/HDL${}^{a}$	3.04	0.82	1.89	5.15	2.82	0.60	2.04	3.72	0.075	0.107	0.489
LDL/HDL${}^{a}$	1.47	0.59	0.77	3.46	1.33	0.43	0.74	1.90	0.102	0.160	0.528
TG/HDL${}^{a}$	0.67	0.58	0.19	2.73	0.55	0.60	0.16	1.99	0.208	0.387	0.596
Males, routine immobilizations only (n=22)											
TC${}^{b}$	6.82	1.85	4.73	12.61	6.58	1.73	3.98	8.35	0.037	0.137	0.792
TG${}^{a}$	1.21	0.55	0.48	3.00	1.06	0.73	0.61	2.35	0.139	0.260	0.600
HDL${}^{b}$	2.50	0.63	1.58	3.70	2.77	1.16	1.18	3.90	-0.265	0.391	0.505
LDL${}^{b}$	3.17	1.30	1.96	7.60	3.08	0.78	2.05	3.93	0.029	0.195	0.881
apoA1${}^{b}$	230.62	59.48	150.37	378.56	247.76	85.40	132.76	344.29	-17.15	33.32	0.612
TC/HDL${}^{a}$	2.77	0.48	1.89	3.45	2.56	0.57	2.04	3.37	0.079	0.093	0.408
LDL/HDL${}^{b}$	1.28	0.35	0.77	2.08	1.25	0.48	0.74	1.79	0.021	0.151	0.892
TG/HDL${}^{a}$	0.52	0.27	0.19	1.20	0.60	0.78	0.16	1.99	-0.144	0.378	0.707
Females, all values retained (n=32)											
TC${}^{a}$	5.86	1.49	4.15	9.41	6.34	1.54	4.84	10.91	-0.078	0.089	0.385
TG${}^{a}$	2.34	2.87	0.69	11.50	1.59	0.93	0.53	3.55	0.389	0.323	0.238
HDL${}^{b}$	1.53	0.65	0.43	2.78	1.88	0.61	0.70	3.13	-0.343	0.225	0.137
LDL${}^{a}$	2.89	1.20	0.79	5.62	3.68	1.67	1.22	7.83	-0.242	0.158	0.136
apoA1${}^{b}$	168.53	68.08	50.87	315.74	204.01	51.12	114.63	299.11	-35.48	21.05	0.102
TC/HDL${}^{a}$	5.10	4.45	1.76	18.77	3.68	1.37	2.11	7.66	0.326	0.224	0.156
LDL/HDL${}^{a}$	2.12	0.96	0.63	3.88	2.22	1.26	0.51	5.14	-0.045	0.189	0.814
TG/HDL${}^{a}$	3.23	7.16	0.27	26.74	1.09	1.04	0.25	3.99	1.08	0.57	0.067
Females, routine immobilizations only (n=25)											
TC${}^{b}$	5.38	0.76	4.15	6.28	6.38	1.67	4.84	10.91	-0.169	0.095	0.087
TG${}^{a}$	1.37	0.77	0.69	3.16	1.42	0.88	0.53	3.55	-0.040	0.250	0.871
HDL${}^{b}$	1.76	0.61	0.68	2.78	1.96	0.52	1.28	3.13	-0.202	0.230	0.388
LDL${}^{b}$	2.81	0.82	1.49	3.82	3.66	1.79	1.22	7.83	-0.266	0.179	0.151
apoA1${}^{b}$	195.07	91.20	92.73	315.74	209.08	50.22	132.69	299.11	-14.01	22.62	0.542
TC/HDL${}^{a}$	3.53	1.82	1.76	8.06	3.36	0.80	2.11	4.76	0.051	0.150	0.739
LDL/HDL${}^{b}$	1.83	0.87	0.63	3.60	1.95	0.88	0.51	3.95	-0.068	0.192	0.726
TG/HDL${}^{a}$	1.09	1.36	0.27	4.65	0.83	0.68	0.25	2.73	0.274	0.412	0.513

${}^{a}$ Distribution of biomarker is left-skewed, differences analyzed using generalized linear models with gamma distribution and log-links. ${}^{b}$ Distribution of biomarker is normally distributed, differences analyzed using linear regression. TC, TG, HDL, and LDL reported in mmol/L; apoA1 reported in mg/dl. apoA1 – apolipoprotein A1; HDL – high-density lipoprotein; LDL – low-density lipoprotein; TC – total cholesterol; TG – triglycerides.

**Table 5:** Differences in lipid markers and lipoprotein ratios in a sample of zoo-housed western lowland gorillas based on presence or absence of cardiac disease.

	Present				Absent				Analysis		
	$\bar{x}$	SD	min	max	$\bar{x}$	SD	min	max	B	SE	p
Both sexes, all values retained (n=61)											
TC${}^{a}$	6.61	1.83	3.81	12.61	6.14	1.45	3.98	10.91	0.074	0.066	0.266
TG${}^{a}$	1.65	2.09	0.48	11.50	1.49	0.89	0.53	3.55	0.106	0.244	0.666
HDL${}^{b}$	2.17	0.80	0.43	3.70	1.92	0.76	0.68	3.90	0.254	0.202	0.213
LDL${}^{a}$	3.18	1.32	0.79	7.60	3.28	1.40	1.22	7.83	-0.032	0.110	0.772
apoA1${}^{b}$	206.74	74.70	50.87	378.56	200.27	59.49	92.73	344.29	6.469	17.18	0.708
TC/HDL${}^{a}$	3.74	3.24	1.76	18.77	3.58	1.39	2.04	8.06	0.044	0.165	0.792
LDL/HDL${}^{a}$	1.62	0.76	0.63	3.88	1.97	1.09	0.51	5.14	-0.197	0.134	0.147
TG/HDL${}^{a}$	1.69	5.14	0.19	26.74	1.07	1.09	0.16	4.65	0.460	0.551	0.407
Both sexes, routine immobilizations only (n=47)											
TC${}^{b}$	6.59	1.80	4.15	12.61	6.21	1.55	3.98	10.91	0.060	0.077	0.441
TG${}^{a}$	1.18	0.54	0.48	3.00	1.38	0.83	0.53	3.55	-0.156	0.161	0.339
HDL${}^{b}$	2.44	0.64	1.58	3.70	2.03	0.76	0.68	3.90	0.405	0.210	0.060
LDL${}^{b}$	3.21	1.25	1.49	7.60	3.29	1.49	1.22	7.83	-0.025	0.126	0.842
apoA1${}^{b}$	231.89	60.01	150.37	378.56	208.24	59.44	92.73	344.29	23.64	17.61	0.186
TC/HDL${}^{a}$	2.77	0.54	1.76	3.50	3.33	1.20	2.04	8.06	-0.186	0.089	0.042
LDL/HDL${}^{b}$	1.37	0.48	0.63	2.27	1.79	0.85	0.51	3.95	-0.269	0.126	0.038
TG/HDL${}^{a}$	0.53	0.28	0.19	1.20	0.90	0.97	0.16	4.65	-0.539	0.261	0.044
Males, all values retained (n=29)											
TC${}^{a}$	6.65	1.91	3.81	12.61	6.36	1.47	3.98	8.35	0.045	0.105	0.670
TG${}^{a}$	1.28	0.65	0.48	3.00	1.02	0.53	0.60	2.35	0.224	0.200	0.271
HDL${}^{b}$	2.32	0.79	0.74	3.70	2.39	0.91	1.18	3.90	-0.067	0.325	0.838
LDL${}^{a}$	3.22	1.24	2.01	7.60	2.90	1.01	1.80	4.85	0.106	0.146	0.473
apoA1${}^{b}$	213.54	72.97	78.71	378.56	226.03	65.87	132.76	344.29	-12.49	27.61	0.655
TC/HDL${}^{a}$	3.06	0.85	1.89	5.15	2.82	0.58	2.04	3.72	0.081	0.100	0.425
LDL/HDL${}^{a}$	1.50	0.61	0.77	3.46	1.28	0.40	0.74	1.90	0.159	0.147	0.290
TG/HDL${}^{a}$	0.69	0.61	0.19	2.73	0.54	0.53	0.16	1.99	0.238	0.359	0.513
Males, routine immobilizations only (n=22)											
TC${}^{b}$	6.93	1.92	4.73	12.61	6.43	1.56	3.98	8.35	0.074	0.122	0.550
TG${}^{a}$	1.22	0.59	0.48	3.00	1.08	0.60	0.61	2.35	0.130	0.231	0.580
HDL${}^{b}$	2.55	0.66	1.58	3.70	2.59	1.00	1.18	3.90	-0.036	0.356	0.920
LDL${}^{b}$	3.27	1.34	2.05	7.60	2.88	0.76	1.96	3.93	0.128	0.170	0.462
apoA1${}^{b}$	234.56	62.38	150.37	378.56	234.43	73.54	132.76	344.29	0.130	30.18	0.997
TC/HDL${}^{a}$	2.76	0.48	1.89	3.45	2.64	0.56	2.04	3.37	0.045	0.085	0.597
LDL/HDL${}^{b}$	1.30	0.36	0.77	2.08	1.22	0.41	0.74	1.79	0.069	0.135	0.618
TG/HDL${}^{a}$	0.52	0.29	0.19	1.20	0.58	0.64	0.16	1.99	-0.109	0.348	0.757
Females, all values retained (n=32)											
TC${}^{a}$	6.48	1.73	4.15	9.41	6.05	1.47	4.31	10.91	0.070	0.106	0.517
TG${}^{a}$	2.68	3.92	0.69	11.50	1.67	0.94	0.53	3.55	0.469	0.353	0.194
HDL${}^{b}$	1.77	0.75	0.43	2.78	1.73	0.62	0.68	3.13	0.037	0.277	0.895
LDL${}^{a}$	3.07	1.63	0.79	5.62	3.44	1.52	1.22	7.83	-0.112	0.198	0.574
apoA1${}^{b}$	188.27	82.04	50.87	315.74	189.96	54.76	92.73	299.11	-1.689	26.17	0.949
TC/HDL${}^{a}$	5.59	6.00	1.76	18.77	3.89	1.51	2.11	8.06	0.364	0.253	0.161
LDL/HDL${}^{a}$	1.94	1.07	0.63	3.88	2.25	1.16	0.51	5.14	-0.150	0.224	0.506
TG/HDL${}^{a}$	4.40	9.86	0.27	26.74	1.27	1.19	0.25	4.65	1.240	0.557	0.034
Females, routine immobilizations only (n=25)											
TC${}^{b}$	5.58	0.86	4.15	6.28	6.13	1.59	4.55	10.91	-0.094	0.122	0.448
TG${}^{a}$	1.06	0.40	0.69	1.56	1.49	0.89	0.53	3.55	-0.342	0.282	0.238
HDL${}^{b}$	2.10	0.48	1.64	2.78	1.84	0.56	0.68	3.13	0.259	0.275	0.357
LDL${}^{b}$	3.02	1.03	1.49	3.82	3.44	1.66	1.22	7.83	-0.130	0.231	0.580
apoA1${}^{b}$	223.88	58.13	168.79	315.74	199.08	52.79	92.73	299.11	24.80	26.88	0.366
TC/HDL${}^{a}$	2.78	0.76	1.76	3.50	3.58	1.28	2.11	8.06	-0.252	0.173	0.158
LDL/HDL${}^{b}$	1.57	0.77	0.63	2.27	1.99	0.88	0.51	3.95	-0.240	0.225	0.298
TG/HDL${}^{a}$	0.55	0.29	0.27	0.95	1.02	1.05	0.25	4.65	-0.619	0.481	0.211

${}^{a}$ Distribution of biomarker is left-skewed, differences analyzed using generalized linear models with gamma distribution and log-links. ${}^{b}$ Distribution of biomarker is normally distributed, differences analyzed using linear regression. TC, TG, HDL, and LDL reported in mmol/L; apoA1 reported in mg/dl. apoA1 – apolipoprotein A1; HDL – high-density lipoprotein; LDL – low-density lipoprotein; TC – total cholesterol; TG – triglycerides.

When all values were retained for multimodel inference, the top model for predicting risk of all-cause morbidity contained age, sex, and LDL, with a 22 % chance ($w_{i}=0.224$) of it being the best model among those tested. All models containing single lipid markers were within the top model set and were within or near ΔAICc < 2 (Table 6). The top model explained 53 % of the variation in morbidity risk (pseudo-$R^{2}$ = 0.527), with the remaining models in the top model set explaining 50 %–53 % (pseudo-$R^{2}$ values range from 0.495–0.525). When only routine immobilizations were included, the top model included age, sex, and LDL/HDL ratio, with a 35 % chance of being the best model ($w_{i}=0.347$). This top model explained 57 % of the variation in risk of all-cause morbidity (pseudo-$R^{2}=0.567$). It was followed by the model containing age, sex, and LDL ($w_{i}=0.169$, pseudo-$R^{2}=0.543$) and the model with only age and sex ($w_{i}=0.143$, pseudo-$R^{2}=0.496$), both of which were within ΔAICc < 2. All except the global model were within the top model set for all-cause morbidity.

**Table 6:** Results from multimodel inference analysis of which models with age, sex, and lipid markers best explain all-cause morbidity risk in zoo-housed western lowland gorillas. Models with weights summing to ≥ 0.95 and Δ AICc < 6 are within the top model set; models in italics are not contained within the top model set.

Model	K	AICc	ΔAICc	$w_{i}$	ER	$R^{2}$
All values retained (n=61)						
Age + sex + LDL	4	61.9	0.00	0.224	–	0.527
Age + sex	3	62.1	0.13	0.210	1.067	0.495
Age + sex +LDL/HDL	4	62.1	0.19	0.205	1.093	0.525
Age + sex + TC	4	63.9	1.99	0.083	2.699	0.501
Age + sex + apoA1	4	64.1	2.18	0.075	2.987	0.498
Age + sex +TG/HDL	4	64.2	2.23	0.068	3.294	0.497
Age + sex + TG	4	64.3	2.40	0.068	3.294	0.495
Age + sex +TC/HDL	4	64.4	2.41	0.067	3.343	0.495
Age + sex + HDL	4	64.4	2.42	0.067	3.343	0.495
*Age*+*sex*+*all*	*10*	*93.9*	*31.96*	*0.000*	-	*0.279*
Routine immobilizations only (n=47)						
Age + sex +LDL/HDL	4	47.7	0.00	0.347	–	0.567
Age + sex + LDL	4	49.1	1.44	0.169	2.053	0.543
Age + sex	3	49.4	1.77	0.143	2.427	0.496
Age + sex +TC/HDL	4	50.3	2.65	0.092	3.772	0.522
Age + sex + TG	4	50.5	2.85	0.084	4.131	0.519
Age + sex +TG/HDL	4	50.7	2.97	0.073	4.753	0.517
Age + sex + TC	4	50.7	3.00	0.077	4.506	0.516
Age + sex + apoA1	4	51.8	4.16	0.043	8.070	0.496
Age + sex + HDL	4	51.8	4.16	0.043	8.070	0.496
*Age* + *sex* + *all*	*10*	*71.1*	*23.47*	*0.000*	–	*0.456*

AICc – Akaike's information criterion corrected for small sample size; ΔAICc – difference between the model with the lowest AICc and the current model; apoA1 – apolipoprotein A1; ER – evidence ratio, weight of the model with the lowest AICc divided by the weight of the current model; HDL – high-density lipoprotein; K – number of variables included; LDL – low-density lipoprotein; $R^{2}$ – coefficient of determination, Nagelkerke's adjusted/pseudo-$R^{2}$; TC – total cholesterol; TG – triglycerides; $w_{i}$ – Akaike's weight, model probabilities.

For risk of cardiac disease, the top model contained age and sex only when all values were retained, with a 32 % chance ($w_{i}=0.319$) of being the best model out of those tested. The rest of the top model set for cardiac disease risk included all models with age, sex, and one lipid marker (Table 7), and all models within the top model set were within or near to ΔAICc < 2, suggesting equivalence with the top model. Variance explained by models within the top model set ranged from 32 %–34 % (pseudo-$R^{2}$ values range from 0.319–0.335). When only routine immobilizations were included, the top model contained age, sex, and TC/HDL ratio ($w_{i}=0.333$, pseudo-$R^{2}=0.431$), followed by the models with age, sex, and TG/HDL ($w_{i}=0.233$, pseudo-$R^{2}=0.427$) and with age and sex only ($w_{i}=0.159$, pseudo-$R^{2}=0.353$). Like all-cause morbidity, the global model was not included in the top model set.

**Table 7:** Results from multimodel inference analysis of which models with age, sex, and lipid markers best explain cardiac disease risk in zoo-housed western lowland gorillas. Models with weights summing to ≥ 0.95 and Δ AICc < 6 are within the top model set; models in italics are not contained within the top model set.

Model	K	AICc	ΔAICc	$w_{i}$	ER	$R^{2}$
All values retained (n=61)						
Age + sex	3	73.1	0.00	0.255	–	0.319
Age + sex +TG/HDL	4	74.2	1.06	0.131	1.947	0.339
Age + sex + TC	4	74.5	1.34	0.130	1.962	0.335
Age + sex + TG	4	74.6	1.46	0.123	2.073	0.333
Age + sex +TC/HDL	4	74.7	1.59	0.115	2.217	0.331
Age + sex +LDL/HDL	4	74.8	1.67	0.111	2.297	0.329
Age + sex + HDL	4	75.0	1.86	0.100	2.550	0.326
Age + sex + apoA1	4	75.3	2.21	0.085	3.000	0.320
Age + sex + LDL	4	75.4	2.29	0.081	3.148	0.319
*Age* + *sex* + *all*	*10*	*92.0*	*18.84*	*0.000*	–	*0.304*
Routine immobilizations only (n=47)						
Age + sex +TC/HDL	4	54.9	0.00	0.333	–	0.431
Age + sex +TG/HDL	4	55.0	0.18	0.233	1.429	0.427
Age + sex	3	56.3	1.47	0.159	2.094	0.353
Age + sex +LDL/HDL	4	56.6	1.79	0.136	2.449	0.396
Age + sex + TG	4	57.4	2.52	0.094	3.543	0.381
Age + sex + apoA1	4	57.5	2.59	0.091	3.659	0.380
Age + sex + HDL	4	57.5	2.64	0.089	3.742	0.379
Age + sex + TC	4	58.7	3.84	0.049	6.796	0.354
Age + sex + LDL	4	58.7	3.86	0.048	6.938	0.353
*Age* + *sex* + *all*	*10*	*70.2*	*15.37*	*0.000*	–	*0.465*

AICc – Akaike's information criterion corrected for small sample size; ΔAICc – difference between the model with the lowest AICc and the current model; apoA1 – apolipoprotein A1; ER – evidence ratio, weight of the model with the lowest AICc divided by the weight of the current model; HDL – high-density lipoprotein; K – number of variables included; LDL – low-density lipoprotein; $R^{2}$ – coefficient of determination, Nagelkerke's adjusted/pseudo-$R^{2}$; TC – total cholesterol; TG – triglycerides; $w_{i}$ – Akaike's weight, model probabilities.

As with cardiac disease, mortality risk was best explained by a top model with only age and sex when all values were retained (Table 8). The top model was only 19 % ($w_{i}=0.190$) likely to be the best model among those included. Consistent with the two previous health outcomes examined, the top model set contained all except the global model and, similar to cardiac disease, these were all within or near ΔAICc < 2. Interestingly, the top model explained the least amount of variation in mortality risk (pseudo-$R^{2}=0.169$), with the rest of the top model set explaining 17 %–21 % (pseudo-$R^{2}$ values range from 0.172–0.212). Results for mortality risk did not change when only values from routine immobilizations were included.

**Table 8:** Results from multimodel inference analysis of which models with age, sex, and lipid markers best explain mortality risk in zoo-housed western lowland gorillas. Models with weights summing to ≥ 0.95 and Δ AICc < 6 are within the top model set; models in italics are not contained within the top model set.

Model	K	AICc	ΔAICc	$w_{i}$	ER	$R^{2}$
All values retained (n=61)						
Age + sex	3	74.3	0.00	0.190	–	0.169
Age + sex +TC/HDL	4	74.4	0.14	0.177	1.073	0.212
Age + sex +TG/HDL	4	74.4	0.18	0.148	1.284	0.212
Age + sex + TC	4	74.6	0.37	0.158	1.203	0.208
Age + sex + TG	4	75.1	0.85	0.124	1.532	0.198
Age + sex + LDL	4	75.2	0.96	0.118	1.610	0.196
Age + sex +LDL/HDL	4	75.5	1.24	0.102	1.863	0.191
Age + sex + apoA1	4	76.4	2.13	0.066	2.879	0.173
Age + sex + HDL	4	76.4	2.17	0.064	2.969	0.172
*Age* + *sex* + *all*	*10*	*90.9*	*16.65*	*0.000*	-	*0.196*
Routine immobilizations only (n=47)						
Age + sex	3	57.2	0.00	0.225	–	0.140
Age + sex + LDL	4	58.0	0.77	0.154	1.461	0.184
Age + sex + TC	4	58.2	0.96	0.139	1.619	0.179
Age + sex + HDL	4	58.7	1.49	0.107	2.103	0.164
Age + sex + apoA1	4	59.2	1.95	0.085	2.647	0.152
Age + sex +LDL/HDL	4	59.2	2.04	0.081	2.778	0.149
Age + sex +TC/HDL	4	59.5	2.28	0.072	3.125	0.143
Age + sex +TG/HDL	4	59.6	2.39	0.068	3.309	0.140
Age + sex + TG	4	59.6	2.39	0.068	3.309	0.140
*Age*+*sex*+*all*	*10*	*76.5*	*19.27*	*0.000*	–	*0.148*

AICc – Akaike's information criterion corrected for small sample size; ΔAICc – difference between the model with the lowest AICc and the current model; apoA1 – apolipoprotein A1; ER – evidence ratio, weight of the model with the lowest AICc divided by the weight of the current model; HDL – high-density lipoprotein; K – number of variables included; LDL – low-density lipoprotein; $R^{2}$ – coefficient of determination, Nagelkerke's adjusted/pseudo-$R^{2}$; TC – total cholesterol; TG – triglycerides; $w_{i}$ – Akaike's weight, model probabilities.

## 4 Discussion

Values reported herein for TC, TG, TC/HDL, LDL/HDL, and TG/HDL (when only routine immobilizations are included) are consistent with data reported from previous studies or in Species360 (ZIMS, 2019), while HDL, LDL, and apoA1 values reported in previous studies or in Species360 (ZIMS, 2019) were higher than values reported in this dataset (Table 1). Older gorillas had significantly higher TG, lower HDL, lower apoA1, and higher TC/HDL, LDL/HDL, and TG/HDL ratios (Fig. 2). In humans, lipid dysregulation increases with age, with increases in total cholesterol, triglycerides, LDL, and lipoprotein ratios having been observed (for review, see Kreisberg and Kasim, 1987). The lack of significant associations of age with TC and LDL in gorillas may be due to their shorter average lifespans compared to humans, as the increases in total cholesterol and LDL with age in the latter happen gradually over multiple decades starting in the third decade of life (Kresiberg and Kasim, 1987). We also observed multiple sex differences, with significantly lower TG, TC/HDL, LDL/HDL, and TG/HDL as well as higher HDL in males, although the relationship between TG and sex was no longer significant when only routine immobilizations were included. We did not find significant differences between males and females for TC or apoA1. Our results are consistent with the only other report to look at age and sex associations with total cholesterol in gorillas, which also showed no significant associations with either variable (McGuire et al., 1989). Sex differences in lipid markers also have been reported in humans. HDL is higher in women than in men (Avogaro et al., 1978; Kang et al., 2011; Kreisberg and Kasim, 1987; Russo et al., 2015; Seidell et al., 1991; Wang et al., 2011), but contradictory sex differences have been reported for total cholesterol (Isles et al., 1989; Kresiberg and Kasim, 1987; Seidell et al., 1991; Weverling-Rijnsburger et al., 1997), triglycerides (Kreisberg and Kasim, 1987; Langsted et al., 2011; Sarwar et al., 2007; Seidell et al., 1991; Wang et al., 2011), LDL (Russo et al., 2015; Wang et al., 2011), and TG/HDL (Gaziano et al., 1997; Kang et al., 2011).

Age and sex were the only significant predictors across models for all-cause morbidity, cardiac disease, and mortality risk, although LDL/HDL ratio did approach significance as a predictor of all-cause morbidity. This is inconsistent with a large body of research in humans, which has repeatedly shown that high levels of TC, LDL, and TG, and high TC/HDL, LDL/HDL, and TG/HDL ratios, as well as low HDL and apoA1, are associated with increased risk of cardiovascular-related morbidity and mortality (Amos et al., 1987; Bittner et al., 2009; Castelli and Anderson, 1986; Chapman et al., 2011; Chen et al., 2020; Corti et al., 1997; Damsgaard et al., 1990; da Luz et al., 2008; Emerging Risk Factors Collaboration, 2009; Gaziano et al., 1997; Hadaegh et al., 2009; Hokanson and Austin, 1996; Horwich et al., 2002; Isles et al., 1989; Iso et al., 1989; Kannel et al., 1971; Kronmal et al., 1993; Langsted et al., 2011; Lemieux et al., 2001; Libby, 2002; Manninen et al., 1992; Millán et al., 2009; Nordestgaard et al., 2007; Ridker et al., 2000, 2001; Sarwar et al., 2007; Schatz et al., 2001; Yunke et al., 2014). Results were similar when we tested multimodel inference as an alternative method for predicting risk, with top model sets for each health outcome including all except the global model. Minimal differences between models within top model sets suggest that while including individual lipid markers alongside age and sex may help predict these health outcomes, they likely do not improve predictions over using age and sex alone. The similarity of results when using multimodel inference compared to more traditional analyses supports the use of these information theoretic approaches instead of, or alongside, null hypothesis significance testing.

The relationship between cardiac disease and LDL in humans is positive (Amos et al., 1987; Emerging Risk Factors Collaboration, 2009; Koba et al., 2002; Lemieux et al., 2001). Given that cardiac disease was by far the most prevalent condition in this sample, the observation that LDL and LDL/HDL ratio, which decreases as LDL declines, may be predictors of all-cause morbidity but not cardiac disease specifically suggests LDL may play a role in other disease processes. With both sexes combined and in females alone, the relationship of LDL/HDL and LDL with all-cause morbidity was negative, meaning gorillas with chronic conditions had lower values than gorillas without. LDL has been observed to be lower in humans with arthritis (Liao et al., 2014; Myasoedova et al., 2010). Arthritis was the second most prevalent chronic condition in our sample, affecting 18 % of individuals (n = 11), and may help explain these results. LDL may even play a causal role as, in humans, those with genetically predicted elevations in LDL are at lower risk for developing arthritis (Hindy et al., 2019). Arthritis has been documented in both captive and wild great apes (Lowenstine et al., 2016), but common risk factors have yet to be investigated. While arthritis is not a major risk factor for mortality, many animals adaptively hide signs of pain or weakness (Markowitz, 1982), which makes diagnosing such conditions difficult for veterinarians. As such, identifying biomarkers to diagnose and monitor arthritis may improve welfare in an aging captive great ape population through the use of medication to reduce pain and inflammation in affected individuals.

Few markers reached or approached significant differences based on presence or absence of chronic conditions and/or cardiac disease. Females with at least one chronic condition approached significantly higher TG/HDL when all values were included and significantly lower TC when only routine immobilizations were included. For cardiac disease, samples collected during routine immobilizations showed higher HDL and lower TC/HDL, LDL/HDL, and TG/HDL ratios in gorillas with cardiac disease, all of which are opposite of what is observed in humans. These results are likely an artifact of the differences between males and females that are observed for these variables, as most are no longer significant when the sample is segregated by sex. However, the difference between TG/HDL in females with and without cardiac disease remains significant and shifts direction, with higher levels in those affected; while this is consistent with human research, the effect is no longer significant when only routine immobilizations are included. Despite LDL/HDL and LDL being possible predictors of all-cause morbidity based on our multimodel inference analyses, they were not significantly different in gorillas based on the presence or absence of chronic conditions. The minimal variation between lipids, lipoproteins, and lipoprotein ratios for gorillas with and without cardiac disease likely reflects differences in etiology. It has recently been suggested that a genetic mutation, estimated to have occurred in a hominin ancestor 2–3 million years ago, may account for the pathological differences in cardiac disease between humans and our closest relatives; loss of cytidine monophosphate-N-acetylneuraminic acid (Neu5Ac) hydroxylase (CMAH) may predispose humans to developing atherosclerotic plaques and CAD, whereas other great apes primarily develop FCM (Kawanishi et al., 2019). However, we again emphasize that it remains to be determined if any differences observed herein are clinically relevant or could be used in any diagnostic capacity.

One limitation of these analyses is the lack of statistical power for including intermediate models (i.e., models containing more than one lipid marker) or interaction terms; small sample size may especially affect multimodel inference analyses (Burnham and Anderson, 2002; Symonds and Moussalli, 2011; Harrison et al., 2018). A second limitation is that while our sample included geriatric females (aged 30–35 years and older), we lack the hormonal and reproductive cycle data necessary to determine menopausal status (Atsalis and Margulis, 2006; Margulis et al., 2007). Menopause can affect circulating lipid levels in women (Treímollières et al., 1999) and so could also play a role in female gorillas. Age cannot be used as a proxy to examine menopausal differences because, as in women, there is considerable variation in timing (Atsalis and Margulis, 2006; Margulis et al., 2007). Given the complex relationship between cardiac disease and metabolic syndrome, glucoregulatory parameters may be valuable in models predicting disease and mortality risk. Glucose, insulin, and insulin resistance were not independently associated with age, suggesting no loss of glucoregulation in older individuals, or with cardiac disease risk in this sample (data not shown). TG/HDL ratio has been used as a “surrogate marker” of insulin resistance in humans (Bittner et al., 2009; Chen et al., 2020; Cordero et al., 2009; Fan et al., 2011), but we observed no significant associations with risk of cardiac disease or other health outcomes in this sample. Research on lipid markers as predictors of disease and mortality in gorillas would further benefit from longitudinal data, as changes in lipid profiles over the lifespan may play a more important role than any single cross-sectional measurement, especially regarding the markers for which we observed significant associations with age. Finally, although we tried to minimize the chances of our results being affected by lipid responses to acute stressors by also analyzing data with immobilizations for non-routine purposes excluded, immobilization alone has been shown to result in increased cortisol in multiple primate species (e.g., olive baboons: Sapolsky, 1982; chimpanzees: Anestis, 2009; Anestis et al., 2006; Lambeth et al., 2006; Whitten et al., 1998; red colobus monkeys: Wasserman et al., 2013; western lowland gorillas: Jacobs et al., 2014) and could potentially trigger fluctuations in lipid markers even in the absence of other stressors. Therefore, the fact that all animals included herein were immobilized may partially explain the lack of differences observed between the two. Furthermore, although it has been suggested that the stress response during immobilization is due to disorientation prior to unconsciousness, rather than the injection itself (Sapolsky, 1982), the method of administration (i.e., hand-injection versus darting) likely impacts perception of the event, which is known to influence physiological responses (Everly and Lating, 2013; Maestripieri and Hoffman, 2011; Ulrich-Lai and Herman, 2009).

Given the paucity of data on lipid markers and lipoprotein ratios in zoo-housed western lowland gorillas, this report contributes to knowledge of both their basic biology as well as factors that may or may not contribute to disease and mortality risk. Our analyses indicate LDL, by itself or within LDL/HDL ratios, may help predict risk of all-cause morbidity when included in predictive models alongside age and sex, with an inverse association observed between LDL and LDL/HDL with disease risk that may reflect high levels of arthritis in the sample. However, using traditional approaches, age and sex best predicted cardiac disease and mortality was best predicted by age alone. When using multimodel inference, models with individual lipid markers were alongside the baseline age and sex model in the top model set, often within ΔAICc < 2. This suggests that while lipid markers may potentially predict cardiac disease and mortality risk in conjunction with age and sex, they are not better at doing so than age and sex alone. Consistent with this suggestion, mean lipid marker values were similar between gorillas irrespective of disease status, with the few significant relationships observed likely being due to differences between males and females. As such, while it remains to be explored whether these results are clinically meaningful, individual lipid markers are unlikely to contribute substantially to efforts to identify biomarkers for predicting and monitoring cardiac disease risk in western lowland gorillas. However, as age and sex fail to explain all observed variability in disease and mortality risk, further investigation into underlying factors is warranted.

## Data Availability

Data supporting the findings of this study are available on request from the corresponding author, ANE, and with agreement from the participating zoological institutions. Individually identifiable data are not publicly available due to confidentiality agreements made between the corresponding author, ANE, and each participating zoological institution.
